# Enhancement mechanism of antioxidant enzyme gene expression by hydrogen molecules

**DOI:** 10.1186/1753-6561-7-S6-P76

**Published:** 2013-12-04

**Authors:** Tomoya Kinjo, Takeki Hamasaki, Hanxu Yan, Hidekazu Nakanishi, Tomohiro Yamakawa, Kiichiro Teruya, Shigeru Kabayama, Sanetaka Shirahata

**Affiliations:** 1 Graduate School of Systems Life Sciences, Kyushu University, Fukuoka 812-8581, Japan; 2 Department of Bioscience and Biotechnology, Faculty of Agriculture, Kyushu University, Fukuoka 812-8581, Japan; 3 Nihon Trim Co. Ltd., Osaka 531-0076, Japan

## Background

Redox regulation system protects our body from oxidative stress-injury and keeps redox homeostasis. The hydrogen molecules (H_2_) exist as stable gas in the ordinal temperature and atmosphere. Recent study reports H_2 _improve ischemia-reperfusion injury, glaucoma, Parkinson's disease and atherosclerosis of animal models. It is supposed from these improvement results that H_2 _participate in reduction of the oxidation stress, however, the reaction mechanism has not been clarified thoroughly. We surmised that intracellular redox regulation system is activated by H_2 _thereupon antioxidative activity is generated. Thus, we tried to find the effect of H_2 _on the Nrf2 pathway, one of the redox regulation systems.

## Materials and methods

HT1080 cells, a human fibrosarcoma cell line, were incubated in a gas incubator at an atmosphere of 75%N_2_/20%O_2_/5%CO_2 _or 75%H_2_/20%O_2_/5%CO_2 _for 24 h. Then, after the cells were treated with H_2_O_2 _or fixative solution for 30 min or 15 min, the intracellular H_2_O_2 _and Nrf2 were determined by In cell analyzer and Confocal laser microscop using a BES-H_2_O_2 _or anti-Nrf2 antibody, respectively. Furthermore, after extraction of mRNA from the treated HT1080 cells, the gene expressions were examined by using Real-time PCR.

## Results

The quantity of intracellular H_2_O_2 _increased by hydrogen peroxide treatment was significantly decreased by pretreatment of H_2_. H_2 _enhanced the expression of catalase, glultathione peroxidase, Cu/Zn-superoxide dismutase, Nrf2 genes and Nrf2 protein.

## Conclusions

It was suggested that H_2 _induced the expression level of antioxidant enzyme genes like catalase and glutathione peroxidase by increasing the expression level of the Nrf2 protein and decreased the amount of intracellular H_2_O_2 _induced by the H_2_O_2 _treatment in HT1080 cells.

